# Conditional Knockout *Kdm2a* Reveals Crucial Involvement in Development and Function of Kidney Collecting Ducts

**DOI:** 10.3390/ijms26031230

**Published:** 2025-01-30

**Authors:** Xianrong Xiong, Hailing Yu, Xupeng Li, Yuan Li, Ruilan Zeng, Yufan Wang, Chunhai Zhang, Yan Xiong, Wei Fu, Honghong He, Shi Yin, Jian Li

**Affiliations:** 1 Key Laboratory for Animal Science of National Ethnic Affairs Commission, Southwest Minzu University, Chengdu 610041, China; 2 Key Laboratory of Qinghai-Tibetan Plateau Animal Genetic Resource Reservation and Exploitation of Ministry of Education, Southwest Minzu University, Chengdu 610041, China

**Keywords:** *Kdm2a*, conditional knockout (cKO), kidney, expression, inflammation

## Abstract

Lysine-specific histone demethylase 2 (*Kdm2a*) is essential for histone modifications involved in development and associated diseases. Nevertheless, the specific functions of *Kdm2a* in renal development and pathology remain largely unexplored. This study aimed to elucidate the roles of *Kdm2a* in sustaining the biological functions of the kidney by generating mutant mice with *Kdm2a* deletion using the *Aqp2*-cre/Loxp system. Our findings showed that *Kdm2a* is widely expressed across various mouse tissues, with particularly high expression in the kidney’s cortex and medulla, surpassing that in other tissues. Despite no observable effects on morphology or survival following the conditional knockout of *Kdm2a*, there was a significant reduction in body weight and bilateral kidney weight compared to controls, most pronounced at the 5-week-old stage (*p* < 0.05). Post *Kdm2a* deletion, kidney metabolic functions were impaired, evidenced by altered levels of creatinine, urea, total cholesterol, and low-density lipoprotein. Histological examination revealed that *Kdm2a*-null kidneys exhibited signs of dysfunction, characterized by macrophage infiltration, fibrosis, inflammatory cell infiltration, and mild thrombosis. Further studies revealed that the expression of chemokine- and pro-inflammatory cytokine-related genes *Il-6*, *Il-8*, *Tnf-a*, and *Il-1β* was significantly increased in the kidneys of Kdm2a cKO mice compared with controls (*p* < 0.05). Additionally, the expression of reabsorption-related genes (*Aqp-3*, *Aqp-5*, and *Aqp-8*) was markedly downregulated in Kdm2a-deficient kidneys compared with controls (*p* < 0.05). Collectively, these findings suggest that *Kdm2a* is crucial for maintaining kidney function and development, partly through the suppression of inflammation and regulation of gene expression. However, the underlying molecular mechanisms of *Kdm2a* in kidney development warrant further investigation.

## 1. Introduction

Renal diseases have emerged as one of the most prevalent healthcare challenges throughout the world, affecting both developed and developing nations. The literature indicates that an increasing number of individuals are grappling with this serious public health issue, which significantly impacts their health and quality of daily life [1,2]. Despite several decades of intensive research and dedicated efforts in nephrology, early prediction, diagnosis, and effective treatment of renal diseases remain challenging [2,3]. Kidney development begins during the embryonic stage and undergoes rapid growth in the initial weeks after birth. Previous studies have revealed that the relative volume and weight of the kidneys are closely associated with their physiological and biological functions [4,5]. The mammalian kidney is composed of numerous nephrons connected by an intricate network of collecting ducts that refine and adjust free water balance, pH levels, and electrolytes to maintain homeostasis within the body [5]. Defects in the collecting duct system usually lead to inflammation and kidney pathology, resulting in renal dysfunction as well as metabolic disorders [5,6,7]. Therefore, a deeper understanding of the mechanism underlying the collecting duct system is essential for preventing, diagnosing, and therapizing kidney diseases. 

Epigenetic modifications, including DNA methylation, histone modifications, and miRNA interference, play critical roles in regulating gene expression. These modifications influence various physiological processes, such as embryonic development, cellular proliferation, aging-related changes, oxidative stress responses, autophagy pathways, and apoptosis [8,9,10]. Growing evidence suggests that epigenetic modification serves as a key pathway for controlling kidney development along with related diseases including inflammation and fibrosis [11,12,13]. Furthermore, epigenetic modification is considered an effective strategy for intervening in kidney diseases [8,9,10]. Notably, epigenetic modifications occur regularly within specific tissues, cell types, or gene-specific contexts and can be reversibly regulated by epigenetic drugs [11,12,13]. Therefore, it is necessary to identify the crucial genes or proteins involved in epigenetic modifications in the kidneys and to elucidate the underlying mechanisms that could enhance clinical treatment strategies for kidney diseases.

Post-translational methylation of histones, particularly the methylation of ubiquitous histones and tissue-specific histone variants, plays a pivotal role in maintaining the normal biological functions within tissues [14,15]. Typically, the steady state of covalent histone methylation is regulated by two groups of enzymes with opposing functions, namely histone demethylase (HDM) and histone methyltransferase (HMT), which remove or add methyl groups to lysine and arginine residues. Lysine-specific demethylase 2a (*Kdm2a*), also known as *Jhdm1a* or *Fbxl11*, belongs to the KDM family of histone lysine demethylase. It contains a catalytic domain known as JmjC and specifically catalyzes the demethylation of H3K36 by binding to the CpG region within gene promoters [16,17]. The growing evidence suggests that *Kdm2a* plays a key role in cell differentiation, apoptosis, and cellular proliferation via executing its H3K36 demethylase activities at target sites [18,19], while dysfunctions associated with *Kdm2a* have been reported across various tumors and cancers [20]. In addition, knockout studies involving *Kdm2a* have exhibited embryonic lethality accompanied by severe growth defects leading to reduced body size [21,22]. Our previous study also demonstrated that *Kdm2a* influences on the transcriptome profile disruptively affect related signaling pathways and alter the epigenetic status of H3K36me during embryonic development, which leads to a compromise in the reproductive performance of both female and male mice [23,24]. However, the expression pattern and precise role of *Kdm2a* in kidney development and function, as well as the underlying regulatory mechanisms governing these processes, have yet to be elucidated. We hypothesized that *Kdm2a* plays an important role during kidney growth and is necessary for maintaining physiological and biological functions in vivo.

In this study, we characterized the spatiotemporal expression profiles and subcellular localization of *Kdm2a* in kidneys. To further investigate the regulatory roles of *Kdm2a* in renal function in vivo, we constructed a model of kidney-specific *Kdm2a* knockout using the collecting duct-specific Cre mouse strain *Aqp2*-Cre. Our findings revealed that both kidney weight and body weight of Kdm2a-null mice were significantly lower than those of controls. Histological analysis indicated that kidney macrophage infiltration and fibrosis were notably increased. Furthermore, loss of *Kdm2a* affected the expression levels of inflammatory factors and reabsorption-related genes in the kidney. These findings provide valuable insights for further investigation into the underlying mechanisms by which *Kdm2a* influences renal development and disease. 

## 2. Results

### 2.1. Expression Pattern and Cellular Localization of Kdm2a

**Figure 1:**
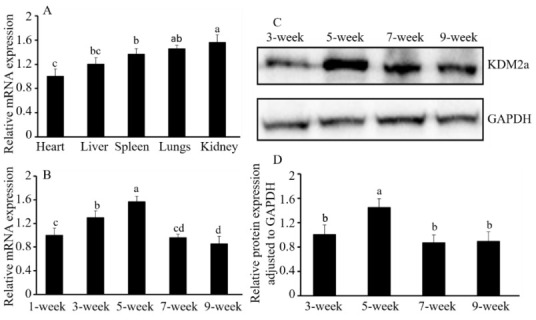
The spatiotemporal expression profile of the *Kdm2a* gene in various tissues and developmental stages of kidneys. (**A**) RT-qPCR analysis showed that the *Kdm2a* gene was significantly expressed in kidneys and lungs of mice (5 weeks old, n = 6). (**B**) RT-qPCR using cDNA of kidneys from 1-week-old, 3-week-old, 5-week-old, 7-week-old, and 9-week-old mice to analyze mRNA expression of *Kdm2a*. (**C**,**D**) Western blotting and quantization showed the expression of Kdm2a protein in kidneys from different stages of wild-type mice. Relative changes in Kdm2a protein during the development of kidneys were presented after normalization with Gapdh. Error bars represent mean ± SEM, and the different superscript letters show significant differences (*p* < 0.05).

To investigate the biological function of *Kdm2a* in the kidneys, we examined its spatiotemporal expression pattern and cellular localization across various mouse tissues. The results showed that *Kdm2a* was expressed in a spatiotemporal manner across multiple tissues, with particularly strong signals in the lungs and kidneys and weaker signals in the heart and liver (Figure 1A). The expression level of *Kdm2a* gradually increased during sexual and body maturation, peaking at 5 weeks old, suggesting a specific role during mouse growth (Figure 1B). However, after the age of 5 weeks, both mRNA and protein abundances of Kdm2a displayed a downward trend (Figure 1B–D).

**Figure 2:**
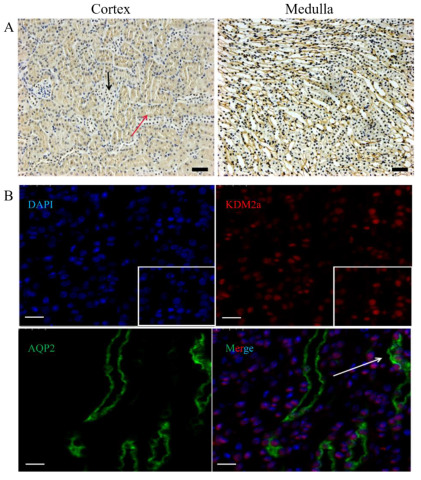
The cellular localization of the *Kdm2a* gene and *Aqp2* in kidneys of 5-week-old mice. (**A**) IHC examined the cellular localization of *Kdm2a* in the cortex and medulla of kidneys from a 5-week-old mouse. (**B**) Co-localization of *Kdm2a* and *Aqp2* via immunofluorescence staining. Blue: DAPI; red: Kdm2a; green: Aqp2. The scale bar: 500 μm.

IHC and fluorescence immunohistochemical analysis were performed to determine the localization of *Kdm2a* in the kidneys. The results showed that *Kdm2a* was ubiquitously expressed in the cortex and medulla, with particularly high expression in the collecting ducts (Figure 2A). Additionally, immunofluorescence also confirmed that Kdm2a was primarily distributed in the cortex and medulla of the kidneys. Interestingly, the localization of AQP2, which is mainly expressed in the collecting ducts, was consistent with previous reports (Figure 2B).

### 2.2. Kdm2a Confirmation of the Deletion of Kdm2a in the Kidney 

**Figure 3:**
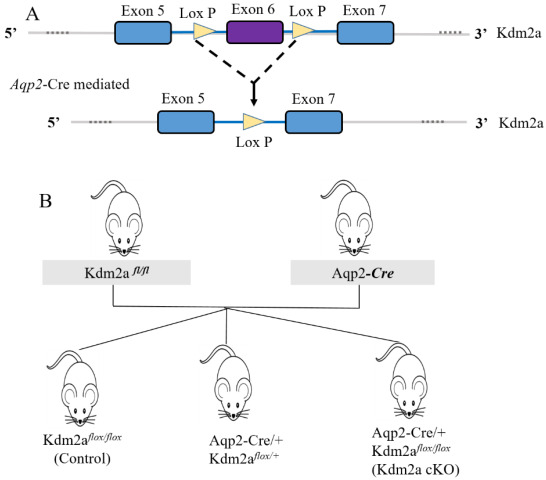
Generation of *Kdm2a* conditional knockout mice. (**A**) The structure of *Kdm2a*, the location of exon 6, and the gene targeting strategy. The *Kdm2a* genomic locus, targeting vector, and targeted allele are sketched and created by insertion of two loxP sites flanking exon 6. (**B**) Breeding strategy to generate *Kdm2a* cKO mice, crossing mice floxed of *Kdm2a* with mice expressing the Cre recombinase under the control of the *Aqp2* promoter, specifically in the collecting duct.

**Figure 4:**
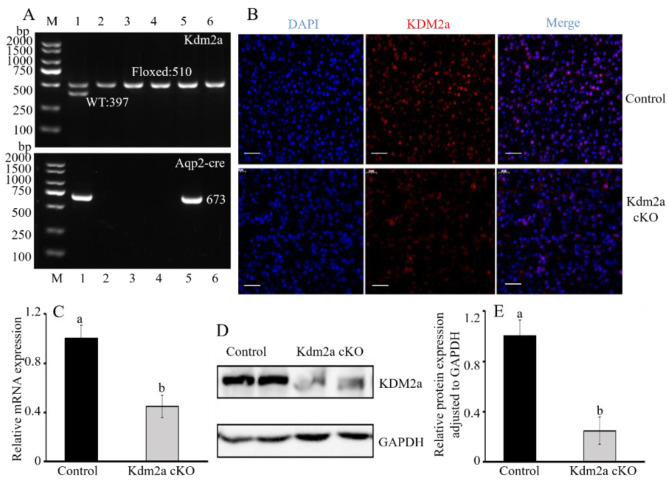
Confirmation of *Kdm2a* conditional knockout mice. (**A**) Genotyping for *Aqp2*-Cre and Kdm2a^flox/flox^. No.1: *Kdm2a*^flox/+^;*Aqp*2-Cre, No.2, 3, 4, 6: *Kdm2a*^flox/flox^ (control), No.5: *Kdm2a*^flox/flox^; *Aqp*2-Cre (*Kdm2a* cKO), M: marker. (**B**) Immunofluorescence staining examined the expression of *Kdm2a* in *Kdm2a* cKO and *Kdm2a*^flox/flox^ kidneys from 5-week-old mice. (**C**) *Kdm2a* mRNA expression in *Kdm2a* cKO and control (*Kdm2a*^flox/flox^) kidneys by RT-qPCR. (**D**) Western blot showed the expression of Kdm2a protein in kidneys from *Kdm2a* cKO and *Kdm2a*^flox/flox^ mice (5 weeks old, n = 6). (**E**) The relative gray of Kdm2a protein between *Kdm2a* cKO and *Kdm2a*^flox/flox^ mice after normalization with glyceraldehyde-3-phosphate dehydrogenase (GAPDH). Data were presented as the mean ± SEM, and the different superscript letters showed significant differences (*p* < 0.05).

To construct a knockout model to examine the functions of the *Kdm2a* gene in kidneys, we generated *Kdm2a* cKO mice by breeding *Kdm2a*-floxed mice with *Aqp2*-Cre transgenic mice. This resulted in frame-shift mutations and the deletion of exon 6 in the collecting duct of the kidney (Figure 3A). The breeding strategy involved crossing *Kdm2a*-floxed mice with *Aqp2*-Cre transgenic mice to obtain *Kdm2a* cKO mice (Figure 3B). The genotype of mutants was validated by genotype PCR, which confirmed the presence of the floxed and Cre bands in the mutants (Figure 4A), identifying them as Kdm2a cKO mice. Immunofluorescence analysis also demonstrated successful deletion of the *Kdm2a* gene (Figure 4B). RT-qPCR analysis showed that approximately 55% of Kdm2a mRNA was lost in the kidneys (Figure 4C). Consistent with these results, western immunoblot analysis also confirmed that Kdm2a was effectively knocked out in the kidneys of Kdm2a cKO mice (Figure 4D,E).

### 2.3. Loss of Kdm2a Affects the Development of Kidney

**Figure 5:**
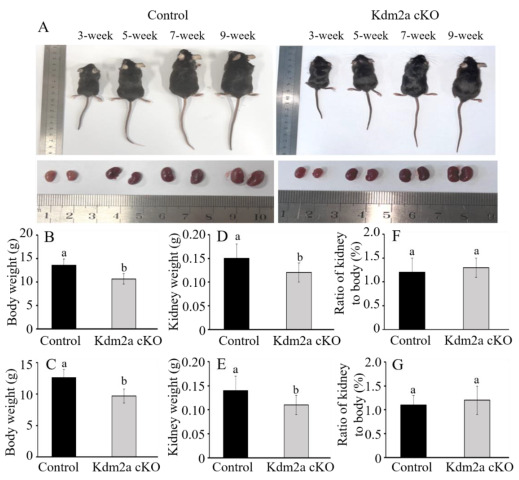
Effects of loss of *Kdm2a* on the development of body and kidneys. (**A**) Individuals of control (*Kdm2a*^flox/flox^) and *Kdm2a* cKO mice at different stages (3 weeks old, 5 weeks old, 7 weeks old, and 9 weeks old). (**B**) The average body weight of the control and Kdm2a cKO female mice at 5 weeks old (n = 6). (**C**) The average body weight of the control and *Kdm2a* cKO male mice at 5 weeks old (n = 6). (**D**) The average kidney weight of the control and *Kdm2a* cKO group female mice at 5 weeks old (n = 6). (**E**) The average kidney weight of the control and *Kdm2a* cKO group male mice at 5 weeks old (n = 6). (**F**) The ratio of kidney weight to body weight of the control and *Kdm2a* cKO female mice at 5 weeks old (n = 6). (**G**) The ratio of kidney weight to body weight of the control and *Kdm2a* cKO male mice at 5 weeks old (n = 6). Error bars represent mean ± SEM, and the different superscript letters showed significant differences (*p* < 0.05).

Kdm2a cKO mice grew normally into adulthood, and no obvious morphological abnormalities were observed compared to age-matched controls (*Kdm2a*^flox/flox^, Figure 5A). We then measured body weights and bilateral kidney weights in Kdm2a cKO and control groups. Our results showed that the body weights of 5-week-old Kdm2a cKO female and male mice were significantly lower than those of their counterparts (*p* < 0.05, Figure 5B,C). Meanwhile, the kidney weights of 5-week-old Kdm2a cKO mice were also notably lighter compared with those of the age-matched controls (*p* < 0.05, Figure 5D,E). Despite this, there was no significant difference in the ratio of the kidney to body weight between Kdm2a cKO and the control groups (*p* > 0.05, Figure 5F,G).

### 2.4. Effects of Kdm2a on the Metabolism of Kidneys

**Figure 6:**
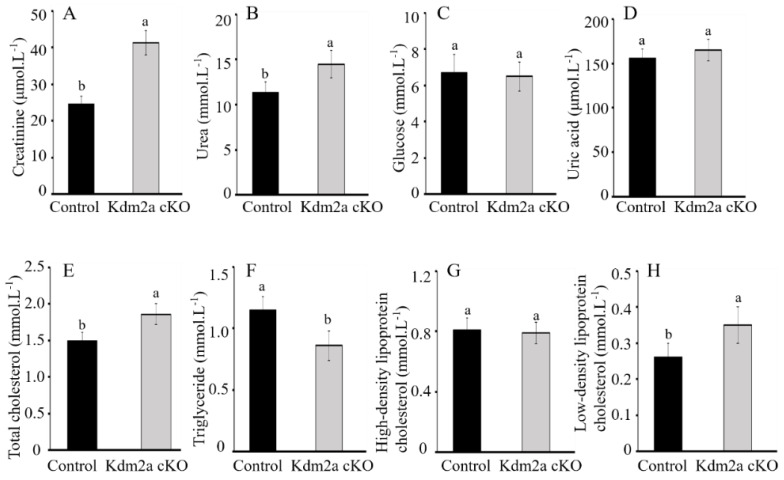
Effects of loss of *Kdm2a* on the metabolic function of the kidneys. The concentrations of creatinine (**A**), urea (**B**), glucose (**C**), uric acid (**D**), total cholesterol (**E**), triglycerides (**F**), high-density lipoprotein (**G**), and low-density lipoprotein (**H**) in the blood were analyzed with a biochemical analyzer (5 weeks old, n = 6). Error bars represent mean ± SEM, and the different superscript letters show significant differences (*p* < 0.05).

To investigate the effects of *Kdm2a* deletion on kidney metabolism, we performed a blood biochemical analysis to assess selected parameters. As shown in Figure 6, there were no statistically significant differences in glucose (6.87 ± 0.41 vs. 6.75 ± 0.38), uric acid (157.66 ± 4.36 vs. 162.29 ± 3.73), or high-density lipoprotein cholesterol (HDL, 0.81 ± 0.29 vs. 0.78 ± 0.32) levels between Kdm2a cKO and control groups (*p* > 0.05). However, Kdm2a cKO had significantly higher levels of urea (14.89 ± 0.92 vs. 11.72 ± 0.78), creatinine (41.87 ± 1.66 vs. 25.23 ± 1.34), total cholesterol (1.92 ± 0.37 vs. 1.51 ± 0.26), and low-density lipoprotein cholesterol (LDL, 0.36 ± 0.14 vs. 0.27 ± 0.08) than those of the control groups (*p* < 0.05). Additionally, the concentration of triglyceride was significantly lower in the blood of Kdm2a cKO mice compared to their counterparts (1.19 ± 0.27 vs. 0.92 ± 0.31, *p* < 0.05). These results suggest that the absence of Kdm2a in the kidneys leads to metabolic disorders in the body.

### 2.5. Kdm2a Deficiency Triggers Severe Inflammation in Kidney

**Figure 7:**
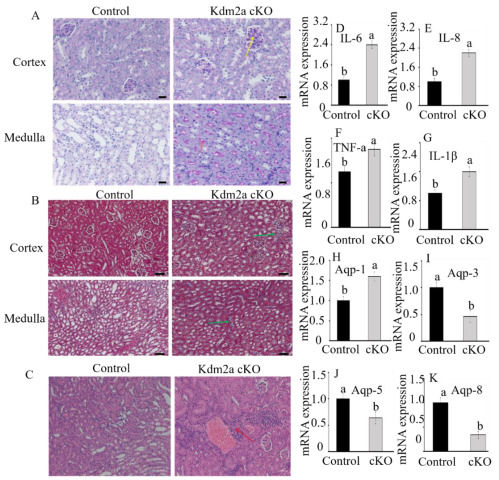
Effects of *Kdm2a* deficiency on the physiological characteristics of the kidney. (**A**) The PAS staining of the kidneys from the control and *Kdm2a* cKO mice at 5 weeks old (n = 6). The yellow arrow represents the glomerular mesangial, and the red arrow points to the collagen fibers. (**B**) The Masson staining of the kidneys from the control and *Kdm2a* cKO mice at 5 weeks old (n = 6). Collagenous material is shown in blue, with cytoplasm and myofibers in red. The scale bar: 50 μm (black). (**C**) The HE staining of the kidney from the control and *Kdm2a* cKO mice at 5 weeks old (n = 6). The red arrow points to the inflammatory factor infiltration. (**D**–**G**) The mRNA relative expression levels of inflammation-related genes (*IL-6*, *IL-8*, *TNF-a*, and *IL-1β*) were detected in the kidneys of the control and *Kdm2a* cKO mice using RT-qPCR (n = 6). Expression levels were normalized to an exogenous control gene (*Gapdh*). (**H**–**K**) The mRNA relative expression levels of reabsorption-related genes (*Aqp-1*, *Aqp-3*, *Aqp-5*, and *Aqp-8*) were detected in kidneys of the control and *Kdm2a* cKO mice using RT-qPCR (n = 6). Expression levels were normalized to an exogenous control gene (*Gapdh*). Error bars represent mean ± SEM, and the different superscript letters showed significant differences (*p* < 0.05).

Inflammation and fibrosis are common pathogenic mechanisms in kidney tissue [25]. To further elucidate the impact of Kdm2a on mouse kidneys, we examined macrophage infiltration in Kdm2a cKO mice. PAS staining revealed a significant increase in macrophage infiltration in the cortex of Kdm2a cKO mice, although this was not significant in the medulla (Figure 7A). We also assessed tubulointerstitial fibrosis, which is a major pathway leading to kidney disease. While wild-type or Kdm2a^flox/flox^ mice did not display obvious signs of fibrosis or tubular atrophy in the respective kidney sections, sections from Kdm2a cKO mice exhibited blue-stained collagen fibers surrounding the to-be or already atrophied tubules in both cortex and medulla (Figure 7B). The HE staining also revealed that inflammatory factor infiltration and mild thrombosis were observed in the section of Kdm2a cKO mice (Figure 7C). 

We further analyzed the expression of chemokine and proinflammatory cytokine genes, which are important for macrophage infiltration in mouse kidneys. We found that the expression levels of the *Il-6* (encoding interleukin-6), *Il-8* (encoding interleukin-8), *Tnf-a* (tumor necrosis factor-alpha), and *Il-1β* (encoding interleukin-*1β*) genes were significantly increased in the kidneys of Kdm2a cKO mice compared to those in their counterparts (*p* < 0.05, Figure 7D–G), consistent with the results of infiltrating macrophages. Taken together, these results demonstrate that Kdm2a deficiency triggers severe inflammation in the kidneys of Kdm2a cKO mice. Furthermore, reabsorption is the main physiological function of the kidneys, especially in the collecting ducts. To further reveal the effects of Kdm2a loss on the function of the kidney, we detected the mRNA expression levels of reabsorption-related genes, including *Aqp-1* (aquaporin-1), *Aqp-3* (aquaporin-3), *Aqp-5* (aquaporin-5), and *Aqp-8* (aquaporin-8). Our results showed that the relative expression abundances of *Aqp-3*, *Aqp-5*, and *Aqp-8* were dramatically downregulated in the kidneys of Kdm2a cKO mice than in those of the control (*p* < 0.05, Figure 7I–K). However, there was a marked upregulation of *Aqp-1* in the Kdm2a cKO group compared with that of the counterparts (*p* < 0.05, Figure 7H).

## 3. Discussion

Although the expression pattern and specific function of *Kdm2a* have been partially elucidated [14,26,27], its physiological and biological functions, especially in kidney development, remain mysterious. In this study, we established the spatiotemporal expression profile and subcellular localization of *Kdm2a* in mouse kidneys. The collecting duct-specific lacking *Kdm2a* model was produced to investigate the biological roles of Kdm2a in the kidneys and the underlying mechanisms. Our main findings demonstrated that *Kdm2a* cKO mice exhibited no significant external abnormalities in appearance, but their body and kidney sizes were on a decreasing trend. Furthermore, histological analysis revealed macrophage infiltration and fibrosis following *Kdm2a* deficiency, accompanied by metabolic dysfunction. This study provides the first in vivo evidence that *Kdm2a* plays an important role in kidney function by regulating body weight, macrophage infiltration, and related gene expression. 

Epigenetic changes are essential for the regulation of kidney development and functions. During this process, key events such as the initiation of transcriptional activity involve a series of epigenetic modifications [28,29]. However, the underlying mechanisms governing the establishment of epigenetic modification during kidney growth, especially histone methylation, remain poorly understood. Therefore, we investigated the spatial and temporal expression profile of Kdm2a, which is a histone lysine-specific demethylase, in the kidney. As a crucial enzyme catalyzing the demethylation of H3K36, Kdm2a contains highly conserved domains (such as JmjC, ZF-CXXC, PHD, and FBOX) and exhibited high evolutionary conservation [30]. It is primarily localized in the nucleus and has ubiquitous expression. Previous studies have shown that Kdm2a is widely expressed in various tissues and stages of embryogenesis, regulating cell proliferation and apoptosis [26,31,32,33]. Given its diverse roles in overall development, it is reasonable to hypothesize that Kdm2a may be important for kidney development. Consistent with previous research [34], we found that Kdm2a was ubiquitously expressed in mice, with the highest abundance in the kidneys. Moreover, Kdm2a mRNA and protein levels were positively correlated with developmental processes in mice, peaking at 5 weeks before showing a downward trend, suggesting a close relationship between Kdm2a and the maturation of individual functions. 

During kidney development, post-transcriptional modifications in histone proteins, especially histone demethylation, play an irreplaceable role in regulating normal morphogenesis and function [35]. Histone demethylation is one of the most extensively studied epigenetic modifications, dynamically controlled by histone-specific methyltransferases and demethylases [36,37]. The appropriate expression of these enzymes is required for proper progression of kidney development [38]. Using the *Aqp-2* Cre/Loxp system, we generated a conditional knockout model that specifically lacked Kdm2a in the collecting ducts. The results showed that there are no significant changes in survival or appearance characteristics of offspring. Our previous study also found that specific knockout of *Kdm2a* in oocytes with *Zp3*-Cre compromised female reproductive ability by arresting the growth and development of follicles and oocytes [23]. Similarly, although *Kdm2a-*deficient males exhibited reduced sperm count and seminiferous tubules density in the testis, accompanied by the spermatogenic degeneration, their fertilization ability and embryonic developmental competence remained comparable to controls [24]. On the contrary, whole genome knockout of *Kdm2a* resulted in early embryonic lethality, accompanied by abnormal brain and tissues development [21,22]. This evidence suggests that *Kdm2a* plays a key role in development, but its effects vary significantly across different tissues or species. Potential explanations for these discrepancies include differences in knockout methods, target sites, and mouse strain, underscoring the necessity for more comparative and functional studies.

Because of the significant roles that histone modifications play in mammalian development, it is reasonable to infer that *Kdm2a* may be crucial for kidney development. Therefore, we utilized Kdm2a cKO mice to further investigate the role of *Kdm2a* in the kidneys. Our results suggested that Kdm2a functions as a regulatory switch that triggers pathological changes and fibrosis by enhancing the mRNA expression of chemokine and proinflammatory cytokine genes during renal development. This finding is consistent with observations in mice with kidney-specific knockout Nrf2 or Ift140, both of which have shown significant effects on inflammation by influencing macrophages and proinflammatory cytokines [39,40]. Inflammation is also an important manifestation of renal pathogenesis. We first examined macrophage infiltration in the kidneys using PAS staining and found that the macrophage infiltration was markedly increased in the cortex after Kdm2a ablation. Masson staining revealed tubulointerstitial fibrosis, characterized by blue-stained collagen fibers in Kdm2a cKO mouse kidneys. In addition, the mRNA expression of chemokine and proinflammatory cytokine genes showed a remarkable increase, consistent with findings in Nrf2-deficient mice [39]. To assess the effects of Kdm2a loss on the function of the kidneys, we evaluated blood biochemical indicators. The levels of creatinine, urea, total cholesterol, and low-density lipoprotein were significantly increased, while triglyceride levels were decreased in Kdm2a cKO mice. High low-density lipoprotein levels in serum are known to induce albuminuria and raise plasma creatinine levels [41,42]. Therefore, kdm2a deficiency in the kidneys likely leads to metabolic dysfunction, which is detrimental to kidney development and exacerbates pathology. 

## 4. Materials and Methods

All animal procedures were conducted in accordance with the guiding principles of the Animals Care and Ethics Committee of Southwest Minzu University (approval number: SMU-CAVS-230211036).

### 4.1. Generation of Kdm2a-Null Mice

Mice were maintained under a 12-hour light/dark cycle with ad libitum access to food and water. The Cre/loxP recombinase system was employed to flank exon 6 of Kdm2a, as detailed in our previous study [23]. To achieve specific deletion of Kdm2a in renal tubular cells, Aqp2-Cre transgenic males were crossed with Kdm2a^flox/flox^ females to produce Aqp2-Cre;Kdm2a^flox/flox^ (named as Kdm2a cKO) offspring. The absence of Kdm2a was confirmed through RT-PCR and primer information provided in the Supplementary Material (Table S1). The Kdm2a cKO mice served as the experimental subjects, while age-matched Kdm2a^flox/flox^ mice served as the controls.

### 4.2. RNA Extraction, RT-PCR, and RT-qPCR

Total RNA was extracted from kidney tissues using Trizol reagent (Invitrogen, Carlsbad, CA, USA) according to the manufacturer’s protocol and methods previously described [43]. Isolated RNA was immediately subjected to reverse transcription using a cDNA synthesis kit (Takara, Dalian, China), following the manufacturer’s instructions. This synthesized cDNA was used as templates for the following PCR analyses. Gene expression levels were quantified using a CFX96 real-time PCR system (BioRad, Hercules, CA, USA), incorporating slight modifications to previously outlined procedures [43]. Briefly, 20 μL of RT-qPCR reaction mixture contained 1 μL of forward and reverse primers, 8 μL of SYBR Green Premix, 2 μL of sample cDNA, and 9 μL of ddH_2_O. Primers were designed for intron-crossing using Primer 5.0 software, and these sequences are listed in the Supplementary Material (Table S2). Relative mRNA expression levels were normalized to the mean abundance of the endogenous control gene glyceraldehyde-3-phosphate dehydrogenase (*Gapdh*) and calculated using the 2^−ΔΔCt^ method. All experiments were performed at least three times.

### 4.3. Assessment of Growth of Kdm2a-deficient Mice

Following genotyping, the mice at different ages (3 weeks old, 5 weeks old, 7 weeks old, and 9 weeks old) of genotypes Kdm2a cKO and Kdm2a^flox/flox^ were weighed and dissected to analyze renal changes. Body weight and kidney weight were recorded, and the ratio of kidney weight to body weight was calculated to evaluate development status.

### 4.4. Immunohistochemistry (IHC) and Fluorescence Immunohistochemical Analysis

Kidneys were collected from 5-week-old mice and fixed in 4% paraformaldehyde overnight at 4 °C. IHC was performed according to routine protocols with minor modification [23]. Sections (5 µm thick) were deparaffinized and rehydrated through a graded ethanol series, followed by antigen retrieval in 10 mM sodium citrate buffer (pH 6.0). Sections were incubated with a primary antibody against Kdm2a (Abcam, ab191387, 1:500) overnight at 4 °C. After washing with PBS, a biotinylated secondary antibody was incubated for 45 min, followed by 3,3′-diaminobenzidine (DAB) and hematoxylin staining. At least three mice per genotype were analyzed. Images were captured using a Zeiss LSM800 confocal microscope (Carl Zeiss, Jena, Germany). 

For fluorescence immunohistochemical analysis, kidney sections prepared as described above underwent incubation with the primary antibody overnight at 4 °C. Slides were then incubated with the secondary antibody and counterstained using DAPI (5 μg/mL in PBS) for 5 min. Immunofluorescence images were obtained utilizing an LSM800 confocal microscope as above.

### 4.5. Western Immunoblots Analysis

Proteins were extracted from the whole kidney tissues as previously described [23]. Briefly, PVDF membranes were incubated with primary antibodies against Kdm2a (Abcam, ab191387, 1:1000) and GAPDH (Abcam, ab26273, 1:1000) overnight at 4 °C. After washing, membranes were incubated with an HRP-conjugated anti-rabbit antibody (bs-40295G-HRP, Abcam) for 2 h at room temperature. Protein bands were visualized using a chemiluminescence detection system (iBright CL100, Thermo, Oklahoma, USA), and protein levels were normalized to the expression of GAPDH. 

### 4.6. Biochemical Assessments of Blood

Plasma biochemical parameters were determined from whole blood samples without anticoagulants at room temperature on a 7080 Biochemical Analyzer (Hitachi High-Technologies, Tokyo, Japan) according to the manufacturer’s instructions. The concentrations of creatinine, urea, glucose, uric acid, total cholesterol, triglycerides (TG), high-density lipoprotein (HDL), and low-density lipoprotein (LDL) in the blood were measured.

### 4.7. Histological Analyses

Fixed-kidney tissues were embedded in paraffin using an automated tissue dehydrator. Paraffin-embedded kidney tissues were sectioned into 5 um thick slices and stained with periodic acid-Schiff (PAS) and Masson’s trichrome staining for morphological analysis. High-power fields containing glomeruli in the cortex and medullary regions were randomly selected from longitudinally sectioned kidney specimens. Cortical and medullary thickness was measured in Masson trichrome-stained sections at comparable areas to evaluate renal fibrosis. Kidney section slides were examined under an LSM800 confocal microscope (Carl Zeiss, Jena, Germany).

### 4.8. Statistical Analysis

Relative fluorescence signals were compared between control and experimental groups using nonparametric tests (Mann–Whitney U-test). Results are presented as mean values ± standard error of the mean (SEM), with *p* < 0.05 considered statistically significant. For multiple mean comparisons, all statistical data underwent one-way analysis of variance (ANOVA) using SPSS 19.0. The fold change in mRNA expression was calculated based on the mean expression values derived from three replicates for each group.

## 5. Conclusions

In summary, we generated mice with collecting duct-specific knockout of *Kdm2a*, which exhibits a kidney-enriched expression profile and spatiotemporal expression during renal development. Although these *Kdm2a* cKO mice were fertile and exhibited no marked morphological or survival abnormalities, their kidneys displayed dysfunction accompanied by macrophage infiltration and fibrosis via regulation of related gene expression. Therefore, we propose that the *Kdm2a* gene plays a crucial role in the kidney function, leading to metabolic disorders and mild inflammation. Further in-depth studies are needed to elucidate the underlying mechanisms of Kdm2a’s role during kidney development. 

## Data Availability

All data is available within the article.

## Supplementary Materials

The following supporting information can be downloaded at https://www.mdpi.com/article/10.3390/ijms26031230/s1
