# Supplementary material for: Conditional Knockout Kdm2a Reveals Crucial Involvement in Development and Function of Kidney Collecting Ducts
Source: Int J Mol Sci. 2025 Jan 30;26(3):1230. doi: 10.3390/ijms26031230 (PMC11818494; doi:10.3390/ijms26031230)
Supplement: Supplementary file 1 [file ijms-26-01230-s001.zip › ijms-3415536-supplementary.pdf]

Table S1 Sequence information of genotyping

Gene name	Primer sequence（5'-3'）	Product length (bp)
Kdm2A$^{flox/flox}$	F: TCACCCTTGCAACTTCCTTCTTCCTT<br>R: TGCACACTGACAAACTCAGCTACCA	floxed: 510 bp<br>WT: 397 bp
Aqp2 Cre	F: CTCTGCAGGAACTGGTGCTGG<br>R: GCGAACATCTTCAGGTTCTGCGG	673 bp

Table S2 RT-qPCR primer sequences

Gene name	Primer sequence（5'-3'）	Tm (°C)
Kdm2a	F: GCCAAGGCACTTGAAAGAAA;<br>R: AGCAGCCTCGAACACTCATT	60
Gapdh	F: CAAGTTCAACGGCACAGTCAA; R:<br>CAAGTTCAACGGCACAGTCAA	60
IL-6	F: TAGTCCTTCCTACCCCAATTTCC;<br>R: TTGGTCCTTAGCCACTCCTTC	60
IL-8	F: CAAGGCTGGTCCATGCTCC;<br>R: TGCTATCACTTCCTTTCTGTTGC	60
IL-1β	F: GCAACTGTTCCTGAACTCAACT;<br>R: ATCTTTTGGGGTCCGTCAACT	60
TNF-α	F: CCCTCACACTCAGATCATCTTCT;<br>R: GCTACGACGTGGGCTACAG	60
Aqp1	F: AGGCTTCAATTACCCACTGGA;<br>R: GTGAGCACCGCTGATGTGA	60
Aqp3	F: GCTTTTGGCTTCGCTGTCAC;<br>R: TAGATGGGCAGCTTGATCCAG	60
Aqp5	F: AGAAGGAGGTGTGTTCAGTTGC;<br>R: GCCAGAGTAATGGCCGGAT	60

Aqp8	F: ATGTGGGAACTCCGGTCCATA;<br>R: ACGGCAATCTGGAGCACAG	60

F: Forward primer; R: Reverse primer.
